# Outcome-Based Medical Education Implication and Opportunities for Competency-Based Medical Education in Undergraduate Pathology

**DOI:** 10.7759/cureus.42801

**Published:** 2023-08-01

**Authors:** Sudarshan Krishnappa, Subhashish Das, Kalyani Raju, Nikhil Chaudhary, Subhashini H Bevinakatti

**Affiliations:** 1 Pathology, Sri Devaraj Urs Medical College, Kolar, IND

**Keywords:** medical graduate, program outcome, course outcome, competencies, curriculum

## Abstract

Introduction

Outcome-Based Education (OBE) is an education model for students that assist the teachers to outline the cause and evaluation with goals in mind to achieve results such as Program Outcome (PO) and Course Outcome (CO) which forms the basis for evaluating student performance.

Materials and methods

This study was conducted with the participants to discuss the hardships faced while implementing Competency-Based Medical Education (CBME). Need-gap analysis based on CBME guidelines was performed. Detailed discussion was done with department faculty to plan.

Results

Internal and final evaluations were done for all the students. When compared with conventional didactic lectures remarkable improvement in academic results of the students were noted which were statistically significant with p value less than 0.001.

Conclusion

CBME is not just adding capabilities but also achieving and strengthening these capabilities with a proper educational approach and efficacious evaluation methods.

## Introduction

To meet global requirements, outcome-based education has become a serious necessity as the traditional method of teaching has several drawbacks and is falling short of expectations. Outcome-Based Education (OBE) is an integrated approach that provides a reforming method for managing medical education to provide proficient learning [1]. Assessment is an integral part of Competency-Based Medical Education (CBME) [2]. The outcomes that are required to be assessed in the CBME curriculum comprise the extent of implementation taking into consideration the coronavirus disease 2019 (COVID-19) pandemic, vertical integral methods, temporal alignment, theory, and practical competencies, the methods of teaching and learning used, the evaluation methods and compliance to the teaching program overall [3]. CBME is an “Outcome-Based Approach” that emphasizes on splitting of competence with learners, especially by taking teaching-learning techniques that keep students as the main focus, inclusive of formative assessment. A published literature search showed health education personnel should methodically assess & subsequently print respective CBME activities inclusive of evaluation material [4]. Hence, the present research aimed to assess mechanisms taken in the execution of CBME in the Second Professional Year in our department. The present research was done in the Department of Pathology. This research study is an attempt to evaluate valuable attributes of the application of OBE at a higher professional educational level in a remote, resource constraint, poverty-stricken rural India.

The main aim was to evaluate the following: 1) To discover possible real difficulties that OBE may face with regard to teaching-learning practices. 2) To assess the execution of OBE evaluation at a higher professional education level. 3) Evaluation of general awareness about OBE. 4) What will be the advantages of adopting OBE.

## Materials and methods

Study design

A qualitative study was done on data collated by focus group discussion (FGD), in-depth interviews (IDI) and checklist-based assessment. 

Inclusion and exclusion criteria

All para-clinical faculty involved in teaching and second-year Bachelor of Medicine, Bachelor of Surgery (MBBS) students were included in the study. The curriculum committee and medical education unit (MEU) members were excluded from the study. 

Sampling and study participants

To recruit subjects, a purposive sampling technique was used. All the faculty of the para-clinical department were involved in the curriculum implementation. A total of 150 second-year MBBS students were assessed.

Data collection methods

The following checklist was utilized for the gap analysis of extracted data. CBME has been introduced recently by the National Medical Commission (NMC), and extensive efforts are needed by the faculties of various medical colleges to implement the same in an effective way. Towards the end (with respect to the proper implementation of the program) our department has undertaken the following steps. These include a) The head of the department and faculty to be sensitized with regards to graduate attributes and learning outcomes of various courses mentioned by regulatory bodies, especially during college council meetings b) Faculty to be trained in framing learning outcomes along with the framing of teaching-learning activities c) Planning of annual teaching schedule stating learning outcomes of the related topics. d) Designing of teaching-learning strategy that enables students to achieve learning outcomes. e) Development of adequate evaluation methods in alignment with learning-teaching methods. In addition following interventions were also done under the guidance of our Medical Education Unit (MOU) i) Competencies were enumerated and specific learning objectives (SLOs) were derived. ii) Lectures were prepared for each competency where all SLOs were included iii) Allotment of teaching hours for each competency. iv) Feasible vertical and horizontal integration methods were analyzed. v) Enumerating demonstrable, observational, assisting, and performance parameters. vii) Planning and conducting Description of a Project (DOAP) sessions. viii) Execution of AETCOM (attitude, ethics, and communications) module. The program supervisor was required to preset and update CO: PO (course outcome: program outcome) mapping in all lesson plans and course outlines before the beginning of a session. With an understanding that all the teaching faculty have a sound knowledge of course content and its CO: PO evaluations, they were asked to give out evaluations and test questions with correct CO: PO. For official documentation purposes, a CO: PO table was made, and marks for every assignment/test were recorded in Microsoft Excel sheets.

## Results

Assessment is the backbone of the CBME curriculum, so it has to be robust and multifaceted. The purpose of assessment is to drive learning and hence is considered indispensable to the learning practice. It provides information to improve instructions (formative assessment) and to measure the achievement of students (summative assessment). Learners securing 50 or more (out of 100 marks combined in theory and clinical) and not <40 marks (in theory and clinical separately) in internal examinations are considered to be eligible for appearing in the University examination. It is mandatory to complete the required certifiable competencies and logbook for that phase of training before appearing in an examination of that subject. Moreover, the students with minimum 75% attendance in theory and 80% in practical/clinical are allowed to appear for the final examination. The University examination theory question paper includes multiple choice questions (not more than 20% of total theory marks), structured essay questions and short answer questions with marks for each part indicated separately. In Pathology, the theory paper and clinical examination are 100 marks each. According to the CBME curriculum, supplementary examination, for those who fail, shall not be conducted later than 90 days from the result declaration date. A student who passes the supplementary examination joins the main batch for progression, and the one who couldn't pass appears in the examination with the junior batch. 

Table 1depicts defined course outcomes for Pathology along with target competency and actual competency attained. Table 1 shows that the actual competency achieved exceeded the target competency.

**Table 1:** Defined course outcome for Pathology along with target competency and actual competency attained PA.CO: Performance assessment-Course outcome

Course outcome	Course outcome description	Target competency (percentage%)	Actual competency (percentage%)	Competency attained?	Remarks
PA.CO1	Describe the causes, evolution, and mechanism of diseases.	50%	68.83%	Yes	
PA.CO2	Describe the alterations in gross and cellular morphology of different organ systems in disease states.	50%	57.33%	Yes	
PA.CO3	Understand the pathological effect of the disease and correlate it with the clinical manifestations and associated complications.	50%	51%	Yes	Students counselled. Faculties and students are sensitized to take remedial and corrective measures.
PA.CO4	Perform and interpret the laboratory investigations necessary for the diagnosis of the disease.	50%	57.66	Yes	

Table 2** **describes the course outcome obtained by the students in the internal and final examinations. A maximum percentage of 68.83% was noted in PA.CO1 and least percentage of 46% was noted in PA.CO3. Table 2 depicts that all the students were evaluated for course outcomes and significant improvement was noted in the overall performance of the students when compared with the internal examination and final examination respectively which were found to be statistically significant (p < 0.001).

**Table 2:** Course outcome obtained by the students in internals and final exams IA: Internal assessment, PA: Performance assessment, CO: Course outcome

Course outcome	Assignment name	Course outcome[CO] attained (percentage%)	Average (percentage%)
PA.CO1	I IA	59%	68.83%
	II IA	92%	
	III IA PAPER 1	44%	
	III IA PAPER 2	68%	
	FINAL PAPER 1	78%	
	FINAL PAPER 2	72%	
PA.CO2	I IA	16%	59.33%
	II IA	70%	
	III IA PAPER 1	71%	
	III IA PAPER 2	52%	
	FINAL PAPER 1	84%	
	FINAL PAPER 2	63%	
PA.CO3	I IA	11%	46%
	II IA	26%	
	III IA PAPER 1	47%	
	III IA PAPER 2	67%	
	FINAL PAPER 1	60%	
	FINAL PAPER 2	65%	
PA.CO4	I IA	55%	57.66%
	II IA	17%	
	III IA PAPER 1	76%	
	III IA PAPER 2	67%	
	FINAL PAPER 1	66%	
	FINAL PAPER 2	65%	

Table 3depicts the comparative statistical analysis of internal examinations and final examinations. A significant improvement was noted in the overall performance of the students when compared with the internal examination and final examination respectively which were found to be statistically significant (p < 0.001).

**Table 3:** Comparative statistical analysis of internal exams and final exams CO: Course outcome, N: Number of students

Comparative statistical analysis of internal exams and final exams						
Course outcome (CO)	Exams conducted	Number of students[N]	Mean	Standard Deviation	t test correlation	p value
CO 1	Internals	141	6.61	2.654	0.481	< 0.001
	Exam	141	22.93	5.315		
CO 2	Internals	141	29.8	6.27	0.522	< 0.001
	Exam	141	17.12	4.093		
CO 3	Internals	141	6.49	3.011	0.332	< 0.001
	Exam	141	24.43	5.48		
CO 4	Internals	140	11.24	2.515	0.297	< 0.001
	Exam	140	16.6	4.208		
Overall performance	Internals	139	54.66	11.654	0.437	< 0.001
	Exam	139	82.15	15.753		

Table 4 demonstrates the CO-PO matrix in accordance with the level of students from beginner to proficient. More students are in the Level 2 phase for all the program outcomes from PO1-PO4.

**Table 4:** Depicts level-wise CO-PO matrix Detailed Report for MBBS - 2019 - Phase II - Pathology  Course Outcome (CO) PO: Program outcome, PA.CO: Performance assessment-Course outcome MBBS 2019- 24 - Course Outcome Level 1          Beginner          0 - 41% Level 2       Developing      41 - 81% Level 3       Proficient         81 - 100%

Competency/CO (Course outcome)	Program outcome PO1 (Percentage%)			Program outcome PO2 (Percentage%)			Program outcome PO3 (Percentage%)			Program outcome PO4 (Percentage%)			Program outcome PO5 (Percentage%)		
	Level 1	Level 2	Level 3	Level 1	Level 2	Level 3	Level 1	Level 2	Level 3	Level 1	Level 2	Level 3	Level 1	Level 2	Level 3
PA.CO1	9.93%	90.07%	-	9.93%	90.07%	-	9.93%	90.07%	-	9.93%	90.07%	-	-	-	-
PA.CO2	16.31%	83.69%	-	16.31%	83.69%	-	-	-	-	16.31%	83.69%	-	16.31%	83.69%	-
PA.CO3	38.30%	61.70%	-	-	-	-	38.30%	61.70%	-	-	-	-	-	-	-
PA.CO4	14.18%	85.82%	-	14.18%	85.82%	-	14.18%	85.82%	-	-	-	-	14.18%	85.82%	-
Average	19.68%	80.32%	0.0	13.48%	86.52%	0.0	20.80%	79.20%	0.0	13.12%	86.88%	0.0%	15.25%	84.75%	0.0

Table 5 depicts deficiencies recognized with regard to the execution of CBME and subsequent action that can be taken for further improvement.

**Table 5:** Deficiencies recognized with regards to the execution of CBME and subsequent action that can be taken ECE: Early Clinical Exposure, MEU: Medical Education Unit, AIT: Alignment and Integration, FDP: Faculty development programs, SDL: Self-Directed Learning, CBME: Competency-Based Medical Education, MEU: Medical Education Units

Themes	Deficiencies recognized	Subsequent action
Active Learning focusing on students Centric Methods	Reinforcement of lecture content in small groups. Efficacious implementation of active learning methods.	Small group teaching only initially at the start of the academic year. MEU workshops to inculcate active learning methods.
Alignment & Integration (AIT)	Objectives were not met, despite sharing them well in advance. Unstructured session for incorporating SDL and assessment in Integration sessions.	Workshops to be conducted, Stringency guidelines to be followed. Designated AIT coordinator should take care of all the aspects. A lesson plan should be shared in advance FDP must be structured as an evaluation with an integration session
Early Clinical Exposure (ECE)	Decreased number of patients	Utilization of other resources such as recorded doctor-patient conversations of crucial cases to sensitize the students. It is suggested to allot faculty as clinical ECE in-charge
Evaluation	Decreased awareness regarding tools needed for formative assessment.	MEU workshops to be conducted to sensitize with regard to formative assessment
Self-Directed Learning (SDL)	Lack of proper protocol for SDL.	MEU workshops to be held

Table 6 shows difficulties confronted by faculty during the execution of CBME such as forming objectives and chalking out a yearly timetable schedule, difficulty in alignment and integration methods, difficulties for the Attitude Ethics and Communication (AETCOM) module, etc.

**Table 6:** Difficulties confronted by faculty during the execution of CBME CBME: Competency-Based Medical Education, COVID-19: Coronavirus disease 2019, MCQ: Multiple choice questions

Themes	Difficulties confronted
To form objectives and chalk out a yearly timetable schedule	Adoption of Curriculum change, Objectives to be framed in accordance with various competencies.
Alignment and Integration	Very hard to align the biochemistry curriculum with pathology. For the preparation of lesson plans for individual Integration sessions will take a longer duration so faculty must be motivated with regard to objectives at the second professional year level.
Attitude Ethics and Communication (AETCOM)	Preparation of the observation guide and analysis of reflection by students was time-consuming. Not able to conduct it during COVID Pandemic.
Skill Modules	Preparation of certification of skill competencies. Not able to conduct it during COVID-19 Pandemic
Assessment	Introduction of MCQs was more challenging in the evaluation as it needs more time consuming work from faculty A structured format for attitudinal evaluation was hard to execute
Self- Directed Learning (SDL)	Lack of clarity in planning train for Self-Directed learning (SDL) and allotment of mentioned hours for the same.

Towards the beginning of the CBME course, there were a lot of apprehensions among the various stakeholders regarding the effectiveness of this new method. However, with the initiation of the program, there was a gradual improvement in perception and increased confidence level among both the faculties and students leading to their enthusiastic participation in the CBME program compared to traditional methods. This is due to various sensitization and orientation programs carried out by the medical education units (MEU) to clear their apprehensions and doubts which ultimately led to better academic performance.

## Discussion

Course outcomes are objectives that students should be able to understand and perform, subsequently, students should declare that he/she had understood and can perform the assigned task. Program outcome concerns with knowledge and skills of medical graduate students [5]. The definition of OBE focuses on students accomplishing their goals with respect to their learning activities. This process requires adhering to the needed outcome like the student should be able to perform a task based on knowledge, ability, and competence until he/she attains an optimum degree of confidence to perform the task independently [6]. These outcomes must be achievable, transparent and should reflect the result of learning. Any assessment process has to assess input and process outcomes. Final conclusion with respect to CBME training can be withdrawn either in the short-term/long term [7]. the short‑term outcome consists of students who depict learning by themselves and clinically capable students that is after completion of their training. For the students who did not acquire competencies, expert faculties should provide positive feedback to them, and subsequently, competency programs should be planned in a systematic way to enhance their performance.

Long‑term outcomes comprise enhanced patient care-related clinical outcomes, needs, and demands of the population and that should match with practical medical training provided [8]. Three basic concepts for OBE: the first concept conveys that success and learning among students are different. More competent teachers understand differences in students' attitudes about teaching and their learning methods. The second concept reflects accomplished learning and encourages more fruitful learning. The third concept conveys that situations that reflect students' learning accomplishments are under the control of teachers and the institute. Above three concepts help in the development of the following four principles of OBE. First, the required learning outcomes must be clear for faculty as well as for students. Second, utilizing the learning outcomes should form the basis for the development of a curriculum according to which teaching methods and evaluation are acquired. Third, students must acquire and learn to sustain themselves under various difficult circumstances. Fourth, providing adequate opportunities to all students helps them to acquire greater performance levels [9]. Execution of a change in the curriculum has to face many obstacles in the way. The major change in teaching-learning methods along with evaluation protocols has been recognized as a daunting task in the current study. Succeeding in the execution of these methods requires coordination among faculty and monetary aid from the administration [10]. 

Shrivastava et al. showed cooperation and coordination with other departments, lack of constant encouragement from the administration, and shortage of trained faculties are likely to affect CBME execution with respect to disorders designated for that session [11]. Assessment plays a major key role and is associated with many internal difficulties. There is a need for trained personnel and sufficient time is needed for its exact execution [12]. Awareness regarding various formative assessment tools was generally lacking among faculty which was the major challenge we faced in the current study. Therefore, NMC advocates that at least one of the assessments must be an assessment regarding the attitude. Pairing assessment with execution can produce indispensable particulars regarding the advancement of the CBME program. So, it is essential to plan an assessment of the execution of the program with the incorporation of changes done along with a review [13]. Although various recommendations are advocated for the fruitful execution of CBME for coming batches. Faculty development acts as a major factor in the implementation of curriculum change. CBME is complex so it needs various reinforcement meetings as faculty development programs (FDPs) to enhance problem-solving aspects in the faculty [14,15].

Limitations of the study

Our study has a few limitations, like it is a unicentric study conducted on a rural resource constraint set-up. Hence incorporation of competencies should be done with frequent hands-on training coupled with appropriate modes of instruction and proper evaluation of feedback mechanisms. This study was not compared with other teaching methods.

## Conclusions

Achieving curriculum changes requires impetus from all collaborators during the execution of the program. Assessment tells about key insights about successes and difficulties for effectuating CBME. CBME is not only about competencies. Competencies are acquired and learned by adequate informational techniques and efficacious evaluation methods. Continuous hands-on training with respect to CBME modules via FDP is a major factor in the fruitful execution of CBME.
